# A Label-Free Liquid Chromatography–Tandem Mass Spectrometry Method for the Quantitative Analysis of Exosome Pharmacokinetics In Vivo

**DOI:** 10.3390/pharmaceutics17060699

**Published:** 2025-05-27

**Authors:** Bingxuan Li, Fei Yu

**Affiliations:** Key Laboratory of Molecular Pharmacology and Drug Evaluation, School of Pharmacy, Ministry of Education, Collaborative Innovation Center of Advanced Drug Delivery System and Biotech Drugs in Universities of Shandong, Yantai University, Yantai 264005, China; libingxuan217@163.com

**Keywords:** exosomes, pharmacokinetics, LC-MS/MS, MRM

## Abstract

**Background:** Exosomes are nanoscale extracellular vesicles actively secreted by cells that play critical roles in disease biomarker discovery, drug delivery, and direct therapeutic applications. However, in vivo pharmacokinetic (PK) studies of exosomes remain limited, hindering their clinical translation. Due to their complex and heterogeneous composition, most existing PK methods rely on chemical or genetic labeling, which may alter their native behavior and complicate accurate analysis. **Methods:** To address this challenge, we developed a label-free liquid chromatography–tandem mass spectrometry (LC-MS/MS) method to investigate the PK of naive exosome-based therapeutic modalities. Exosomes were isolated from rat plasma using size exclusion chromatography (SEC) and quantified using multiple reaction monitoring (MRM) targeting specific exosomal peptides as surrogate analytes. Following intravenous administration of exosomes via the tail vein, plasma concentrations were determined by peptide peak areas, and PK parameters were calculated using a non-compartmental model. **Results:** The method was rigorously validated for specificity, linearity, sensitivity, and reproducibility. Its feasibility was further confirmed by successfully characterizing the PK profile of HEK 293F-derived exosomes in rats. **Conclusions:** This analytical strategy enables direct, label-free quantification of exosomes in plasma and provides a robust platform for advancing exosome-based drug development and translational research.

## 1. Introduction

Exosomes are lipid bilayer-enclosed extracellular vesicles secreted by virtually all cell types under physiological conditions. Typically ranging from 40 to 160 nm in diameter, they encapsulate a diverse array of proteins, nucleic acids, and lipids derived from their cells of origin. Exosomes perform vital biological functions and have shown considerable promise in clinical applications [1,2]. Their functional roles are largely attributed to the bioactive cargo they carry, which can be transferred to recipient cells to facilitate intercellular communication [1,3]. Due to their presence in various body fluids and their molecular signatures reflective of their parent cells, exosomes are increasingly recognized as valuable biomarkers for disease diagnosis, prognosis, and therapeutic monitoring [1,4]. Their involvement in numerous physiological and pathological processes—particularly in immune modulation and tissue regeneration—endows them with intrinsic therapeutic potential [1,5]. Extensive studies have highlighted their efficacy in the treatment of a wide range of diseases, including cancers, cardiovascular disorders, neurodegenerative conditions, and inflammatory diseases [6,7,8,9,10,11,12]. Moreover, exosomes are emerging as highly promising drug delivery vehicles. They can transport proteins, nucleic acids, and small-molecule drugs with remarkable efficiency. Compared to conventional nanocarriers, such as liposomes, micelles, and polymeric nanoparticles, exosomes offer distinct advantages, including lower immunogenicity, minimal toxicity, and the unique ability to cross the blood–brain barrier—factors that enable safer and more effective therapeutic delivery [13,14,15]. Notably, native exosomes have been shown to possess inherent tumor-targeting capabilities [16]. Leveraging exosomes to enhance antitumor immunity and deliver therapeutic agents holds great potential for advancing cancer immunotherapy [17].

To elucidate the biological functions of exosomes and fully harness their therapeutic potential, a comprehensive understanding of their pharmacokinetic (PK) is essential. However, investigating exosome PK in vivo presents substantial challenges. First, since nearly all cells secrete endogenous exosomes, it is difficult to distinguish exogenously administered exosomes from their endogenous counterparts, thereby complicating the tracking of their biodistribution and metabolic fate. Furthermore, current exosome isolation techniques may fail to completely eliminate similarly sized extracellular vesicles or protein aggregates, which can introduce sample impurities and compromise the accuracy of experimental results [18].

Currently, three primary strategies are employed for in vivo exosome PK studies. The first involves radiolabeling exosomes with radioactive tracers for nuclear imaging. While this method provides high sensitivity and labeling efficiency, enabling detection in deep tissues, it carries inherent safety concerns and suffers from limited spatial resolution [19,20]. The second strategy utilizes genetic engineering to transfect donor cells with fluorescent proteins fused to exosomal membrane proteins, enabling bioluminescent imaging. Although this method facilitates high-sensitivity and relatively high-throughput tracking, issues such as uneven substrate distribution, suboptimal emission wavelengths, and potential cytotoxicity limit its broader applicability [21]. The third approach relies on lipophilic dyes to label exosomes for optical imaging. This technique is widely used due to its ease of application and strong fluorescence signal; however, it suffers from critical drawbacks, including dye aggregation that mimics exosomes in size—resulting in false positives—and dye persistence that may not accurately reflect exosome clearance or biodistribution [19,22,23].

Although each of these approaches has advantages and continues to provide valuable insights into exosome PK, they all rely on exosome modification or labeling. These alterations can fundamentally change the composition or behavior of exosome-based therapeutics, potentially skewing the interpretation of their true PK. Therefore, the development of a non-invasive, label-free method is critically needed to enable accurate and physiologically relevant assessment of exosome PK in vivo. Given the complex molecular composition of exosomes and the presence of abundant endogenous vesicles, detecting unmodified exosomes in vivo without prior labeling or engineering remains a significant analytical challenge. Exosomal proteins, distributed across the membrane and lumen, are among the most abundant and conserved components of exosomes [24]. To address this, we selected specific proteolytic peptides derived from exosomal proteins as surrogate markers. By exploiting the linear correlation between exosome concentration and the peak area of these peptides, we developed an MRM-based LC-MS/MS method for quantifying exosome concentrations in plasma without requiring any modification of the vesicles.

MRM is a targeted mass spectrometry technique that detects analytes based on predefined molecular characteristics, allowing for protein quantification via specific proteolytic peptides [25]. In MRM mode, the instrument monitors unique precursor/product ion pairs in real time, each corresponding to a distinct peptide or protein. This dual-stage filtering substantially reduces interference from co-eluting species, suppresses background noise, and enhances signal intensity. Consequently, trace-level analytes—either known or hypothesized—can be measured with high selectivity and precision. The method offers a dynamic range exceeding four orders of magnitude and high sensitivity, enabling the antibody-free detection of proteins and their post-translational modifications [26]. Currently, the most widely adopted strategy in targeted proteomics involves the indirect quantification of proteins through their signature peptides generated by enzymatic digestion, most commonly via LC-MS/MS [27].

In this study, we focused on exosomes derived from HEK 293F cells. HEK 293F, a human embryonic kidney-derived cell line, is widely employed due to its high transfection efficiency, rapid proliferation, and capability to grow in serum-free suspension cultures. Since 2015, seven therapeutic products derived from HEK cells have received FDA approval [28]. HEK 293F exosomes were administered intravenously to rats, and plasma samples were collected at predetermined time points. Exosomes were isolated using size exclusion chromatography (SEC), followed by denaturation, reduction, alkylation, and tryptic digestion to generate peptides. The plasma concentrations of exosomes were then determined by quantifying three exosome-specific peptides using LC-MS/MS based on peak area integration.

## 2. Materials and Methods

### 2.1. Main Instruments

The main instruments employed in this study included the ACQUITY UPLC H-Class ultra-high-performance liquid chromatography (UHPLC) system (Waters Corporation, Milford, MA, USA), the 6500 QTRAP mass spectrometer (AB Sciex, Framingham, MA, USA), an ultracentrifuge (Thermo Fisher Scientific, Waltham, MA, USA), and the NanoSight NS300 nanoparticle tracking analyzer (Malvern Panalytical, Malvern, UK).

### 2.2. Chemicals

Standard 293F-derived exosomes and the Exosupur™ Exosome Purification Kit were obtained from Echo Biotech (Beijing, China). HPLC-grade acetonitrile was purchased from Merck (Darmstadt, Germany). Formic acid, calcium chloride, dithiothreitol (DTT), Tris base, iodoacetamide (IAM), and guanidine hydrochloride were all obtained from Sigma-Aldrich (St. Louis, MO, USA). Magnetic beads conjugated with anti-CD63, anti-CD81, and anti-CD9 antibodies were supplied by Thermo Fisher Scientific (Waltham, MA, USA). The Enhanced BCA Protein Assay Kit, SDS-PAGE Running Buffer, and Western Transfer Buffer were purchased from Beyotime Biotechnology (Shanghai, China). Both primary and secondary antibodies were obtained from Abcam (Cambridge, UK).

### 2.3. Experimental Animals

Male Sprague Dawley rats, weighing approximately 220 g, were procured from Jinan Pengyue Experimental Animal Breeding Co., Ltd., based in Jinan, China. All tested rats were housed under standard laboratory conditions with suitable room temperature and relative humidity. All procedures involving experimental animals were approved by the Experimental Animal Ethics Committee of the College of Pharmacy, Yantai University. (No. 20240224).

### 2.4. Characterization of Exosome

The purchased exosomes were characterized using multiple techniques. Total protein concentration was determined using the Enhanced bicinchoninic acid assay (BCA) Assay Protein Assay Kit according to the manufacturer’s instructions. For nanoparticle tracking analysis (NTA), exosome samples were diluted 1:5000 in phosphate-buffered saline (PBS), and measurements were performed using the NanoSight NS300 under the following settings: camera level 14 and detection threshold 5. Data analysis was conducted using NTA Software version 3.4. Western blotting (WB) was used to detect exosome-specific surface markers. Exosome samples containing 10 μg of protein were separated by SDS-PAGE at 120 V and transferred onto polyvinylidene fluoride membranes at 100 V. The membranes were blocked with quick blocking buffer and then incubated overnight with primary antibodies against exosome markers. The following day, the membranes were washed and incubated with appropriate secondary antibodies for 4 h. After final washes with tris-buffered saline with tween-20 (TBST), the membranes were developed for signal visualization.

### 2.5. Selection of Signature Peptides for LC-MS/MS Analysis

To identify candidate signature peptides for LC-MS/MS analysis, 11 exosome-associated proteins were selected from the top 100 exosomal proteins listed in the EVpedia database (https://evpedia.info/evpedia2_xe/ (accessed on 30 June 2024)). These included CD63, CD9, CD81, TSG101, Cofilin-1, ALIX, HSP70, FLOT2, CHMP6, GAPDH, and FLOT1. Protein sequence data were obtained from UniProt (www.uniprot.org (accessed on 15 July 2024)) and imported into Skyline-daily software for in silico tryptic digestion and peptide prediction.

The specificity of candidate peptides was assessed using the BLAST tool (https://blast.ncbi.nlm.nih.gov/Blast.cgi (accessed on 21 July 2024)) to ensure sequence uniqueness. Subsequently, HEK 293F-derived exosome standards and blank rat plasma samples were digested with trypsin, and LC-MS/MS analysis was performed to experimentally validate the presence and specificity of the predicted peptides.

### 2.6. Isolation of Exosomes from Rat Plasma

Three exosome isolation techniques—SEC, ultracentrifugation (UC), and immunomagnetic adsorption (IMA)—were evaluated for their efficacy in isolating exosomes from rat plasma. To facilitate comparative analysis, 200 μL of plasma was spiked with a defined quantity of exosome standards (with known particle counts) prior to isolation. Each method was subsequently applied to enrich exosomes from the spiked samples. The quantification of the recovered exosomes was performed using NTA, and the optimal method was selected based on particle yield and reproducibility.

#### 2.6.1. UC

Plasma samples were centrifuged at 10,000× *g* for 20 min at 4 °C to remove debris. The supernatant was filtered through a 0.22 μm sterile membrane and then subjected to ultracentrifugation at 135,000× *g* for 70 min at 4 °C. The resulting pellet was resuspended in PBS and ultracentrifuged again under identical conditions. The final exosome pellet was resuspended in 50 μL of PBS, transferred to cryovials, and stored at −80 °C for further use.

#### 2.6.2. SEC

Plasma exosomes were isolated using Exosupur™ SEC columns (Echo Biotech Co., Ltd., Beijing, China) following the manufacturer’s protocol. The columns demonstrated an elution rate of 2 min and 35 s per 200 μL, with an effective size separation range of 30–150 nm and a void volume of 600 μL Prior to sample loading, each column was equilibrated with 10 mL of PBS at room temperature. A 200 μL plasma sample was then applied to the column and allowed to enter completely. Subsequently, 600 μL of PBS was added to initiate elution, and the effluent was collected in 200 μL fractions. The first three fractions were discarded. After adding an additional 1 mL of PBS, fractions 4 to 8 were collected, concentrated to 50 μL using ultrafiltration tubes, and stored at −80 °C for subsequent analysis.

#### 2.6.3. IMA

Totals of 50 μL each of CD63-, CD9-, and CD81-conjugated magnetic beads were added to the plasma samples. The mixture was incubated overnight at 4 °C on a rotary shaker to facilitate antibody-mediated capture of exosomes. The samples were then placed on a magnetic rack, and the supernatant was carefully removed. Bead–exosome complexes were eluted with 50 μL of a 2% SDS-containing buffer to release the exosomes. The supernatant containing the isolated exosomes was collected, aliquoted into cryovials, and stored at −80 °C for further analysis.

### 2.7. Sample Preparation for Mass Spectrometry Analysis

Exosome samples and blank plasma samples isolated by SEC were subjected to proteolytic digestion for LC-MS/MS analysis. Briefly, 6 M guanidine hydrochloride was added to the samples and vortexed thoroughly to ensure complete denaturation. Subsequently, 25 mM DTT was added, and the samples were incubated at 55 °C for 30 min to reduce disulfide bonds. After cooling to room temperature, 50 mM IAM was added for alkylation, and the mixture was incubated in the dark at room temperature for 40 min.

### 2.8. LC-MS/MS

A 10 μL aliquot of the processed sample was injected into an ACQUITY UPLC H-Class Ultra-Performance Liquid Chromatography system. Chromatographic separation was performed using an ACQUITY UPLC CSH C18 column (1.7 μm, 2.1 mm × 150 mm) maintained at 60 °C, with a flow rate of 0.3 mL/min. The mobile phase consisted of (A) 0.1% formic acid in water and (B) acetonitrile. A gradient elution was applied as follows: 2% to 35% B from 4 to 70 min, ramped to 80% B from 70 to 76 min, and then returned to 2% B from 76 to 85 min. The total run time was 85 min. The autosampler temperature was maintained at 4 °C. Mass spectrometric analysis was performed on a Triple Quad 6500 mass spectrometer equipped with a positive electrospray ionization (ESI) source. The instrument was operated with the following parameters: curtain gas (gas 1), 40 psi; auxiliary gas (gas 2), 40 psi; ion spray voltage, +5500 V; and source temperature, 500 °C.

Data acquisition was carried out in MRM mode. Quantifier ions and transitions were selected and optimized using the open-source software Skyline-daily.

### 2.9. Preparation of Calibration Standards and QC Samples

HEK 293F-derived exosome standard samples were used to quantify total exosome particle concentrations in rat plasma. The exosome standards were serially diluted in PBS to the following concentrations: 1.2 × 10^11^, 6.0 × 10^10^, 3.0 × 10^10^, 1.2 × 10^10^, 6.0 × 10^9^, 1.2 × 10^9^, and 6.0 × 10^8^ particles/mL for P1/P2 and 1.2 × 10^11^, 6.0 × 10^10^, 3.0 × 10^10^, 1.2 × 10^10^, 6.0 × 10^9^, 1.2 × 10^9^, and 3.0 × 10^8^ particles/mL for P3. For each concentration level, 100 μL of the diluted exosome standard was mixed with 100 μL of blank rat plasma to prepare calibration standards with final concentrations of 6.0 × 10^10^, 3.0 × 10^10^, 1.5 × 10^10^, 6.0 × 10^9^, 3.0 × 10^9^, 6.0 × 10^8^, and 3.0 × 10^8^ particles/mL for P1/P2 and 6.0 × 10^10^, 3.0 × 10^10^, 1.5 × 10^10^, 6.0 × 10^9^, 3.0 × 10^9^, 6.0 × 10^8^, and 1.5 × 10^8^ particles/mL for P3.

Quality control (QC) samples were prepared in a similar manner at low, medium, and high concentration levels: 9.0 × 10^8^, 6.0 × 10^9^, and 5.0 × 10^10^ particles/mL for P1/P2 and 3.0 × 10^8^, 6.0 × 10^9^, and 5.0 × 10^10^ particles/mL for P3, respectively.

### 2.10. Assay Validation and Sample Analysis

The quantification of exosome concentrations in rat plasma using LC-MS/MS was validated in accordance with the ICH M10 guidelines (European Medicines Agency, EMA). The validation parameters included specificity, linearity, lower limit of quantification (LLOQ), stability, accuracy and precision, extraction recovery, and matrix effects. For each analytical batch, the acceptance criteria for both calibration standards and QC samples were set at ±15% of the nominal value, with a ±20% deviation permitted at the LLOQ.

### 2.11. Sample Collection and Analysis

As no standardized protocols currently exist for the administration of exosomes in animal models, we referenced published literature and administered 1 mL of HEK 293F-derived exosomes (3 × 10^11^ particles) via intravenous tail vein injection into three SD rats. The animals were fasted for 12 h prior to injection, with free access to water.

Post-administration, approximately 0.6 mL of blood was collected at predetermined time points (5, 10, 15, and 30 min; 1 and 2 h) via the orbital venous plexus. Blood samples were transferred to EDTA-coated tubes, centrifuged to separate plasma, and stored at −80 °C until analysis.

Exosomal peptide peak areas were extracted using Skyline software (https://skyline.ms/project/home/begin.view?/ accessed on 21 April 2025). The exosome particle concentrations at each time point were calculated based on the standard curves derived from three signature peptides. PK parameters were determined by analyzing plasma concentration–time profiles using PKSolver V2.0. Exosome concentration–time curves were generated with Origin 2024 software.

## 3. Results

### 3.1. Isolation of Exosomes from Rat Plasma

The purchased HEK 293F-derived exosomes were characterized to confirm their typical exosomal properties (Supplementary Figure S1), validating their suitability for subsequent experimental procedures. Exosomes isolated from rat plasma using three different methods—SEC, UC, and IMA—were evaluated for particle yield and purity.

**Figure 1:**
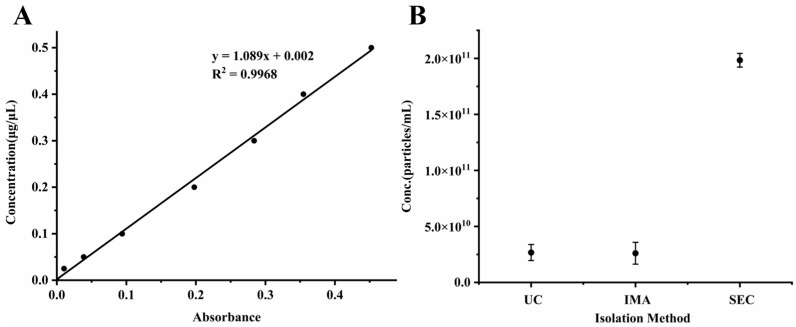
(**A**) Standard curves generated for total protein concentration determination using the BCA across the three exosome isolation methods: UC, IMA, and SEC. (**B**) Comparison of exosome particle concentrations isolated from rat plasma using three methods, as determined by NTA.

Total protein content was assessed using the BCA (Figure 1A), while NTA was performed to determine exosome particle counts (Figure 1B). Particle count data provided a direct comparison of the isolation efficiency among the three methods. In contrast, total protein concentration served as an indicator of co-isolated plasma protein contamination.

Among the tested methods, SEC yielded the highest number of exosome particles. UC and IMA demonstrated similar particle yields but were associated with significantly higher levels of contaminating plasma proteins (Table 1). Comparative analysis of the three methods highlighted SEC as the most effective approach for isolating plasma-derived exosomes in this study, offering an optimal balance between isolation efficiency and sample purity.

### 3.2. Selection of Signature Peptides for LC-MS/MS Analysis

Signature peptides for 11 exosomal proteins were initially predicted using Skyline software and subsequently evaluated for sequence specificity using BLAST (https://blast.ncbi.nlm.nih.gov/Blast.cgi (accessed on 21 July 2024)). The peptide sequences confirmed as unique by BLAST analysis are listed in Table S1. Trypsin-digested samples of HEK 293F exosome standards and blank rat plasma were then subjected to LC-MS/MS analysis. By comparing sequence specificity and chromatographic peak area responses, three signature peptides corresponding to three exosomal proteins were conclusively selected:

ALIX: TMQGSEVVNVLKSLLSNLDEVKK (designated as P1)

FLOT1: LAEAEISSQLIMQAEAEAASVRMR (designated as P2)

HSP70: AFYPEEISSMVLTK (designated as P3)

Detailed peptide information identified from HEK 293F exosome standards via LC-MS/MS is provided in Table S2.

### 3.3. Method Validation

#### 3.3.1. Selectivity and Specificity

**Figure 2:**
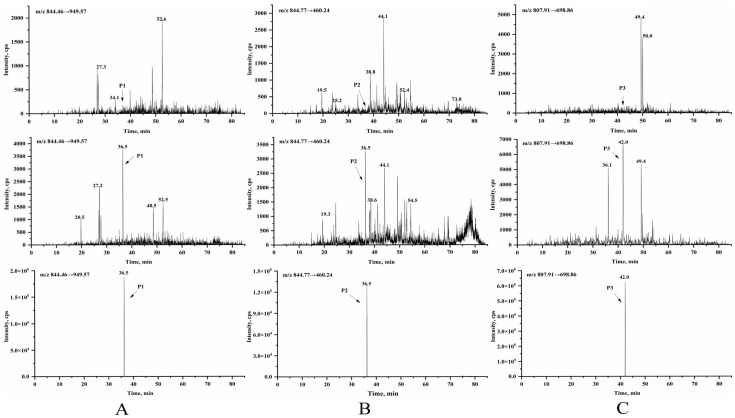
Representative chromatograms of signature peptides in rat plasma: (**A**) P1 (*m*/*z* 844.46 → 949.57), (**B**) P2 (*m*/*z* 844.77 → 460.24), and (**C**) P3 (*m*/*z* 807.91 → 698.86). From top to bottom: blank rat plasma, plasma spiked with exosomes at the LLOQ, and plasma collected 5 min after intravenous administration of exosomes.

Representative chromatograms of blank plasma, blank plasma spiked with exosomes at the LLOQ, and a rat plasma sample collected 5 min after intravenous exosome administration are shown in Figure 2. No interfering peaks were observed at the retention times corresponding to the signature peptides P1, P2, and P3 in the blank plasma samples, confirming the method’s specificity.

#### 3.3.2. Linear Range

Linear regression analysis using the least squares method was conducted between the peak areas of the three signature peptides and corresponding exosome particle concentrations (as shown in Table 2). For peptides P1 and P2, a linear quantification range of 3.0 × 10^8^ to 6.0 × 10^10^ particles/mL was established, with an LLOQ of 3.0 × 10^8^ particles/mL. For peptide P3, the linear range extended from 1.5 × 10^8^ to 6.0 × 10^10^ particles/mL, with an LLOQ of 1.5 × 10^8^ particles/mL.

#### 3.3.3. Accuracy and Precision

The precision and accuracy of the assay were evaluated using low, medium, and high-concentration QC samples of exosomes in rat plasma, as summarized in Table 3. Both intra-day and inter-day accuracy, expressed as relative error (%RE), and precision, expressed as coefficient of variation (%CV), were within the acceptable limits defined by regulatory guidelines—±20% for the LLOQ and ±15% for other QC levels. These results demonstrate that the developed LC-MS/MS method is reliable and reproducible for the quantitative analysis of exosomes in plasma samples.

#### 3.3.4. Matrix Effects and Method Recoveries

Extraction recovery was evaluated by comparing the peak areas of low, medium, and high-concentration QC samples with those of post-extraction spiked samples at corresponding concentrations. For peptide P1, recovery rates ranged from 89.2% to 97.5%; for peptide P2, from 89.0% to 94.4%; and for peptide P3, from 86.6% to 98.9%. These results indicate that the sample preparation and analytical procedures are consistent and reproducible. Detailed recovery data are provided in Table 4.

Matrix effects were assessed by calculating the ratio of the peak area of analytes spiked into blank plasma extract (matrix) to that in the pure solution (no matrix), across the three QC levels. For peptide P1, matrix effect values ranged from 96.0% to 108.6%; for peptide P2, from 89.1% to 98.6%; and for peptide P3, from 94.1% to 102.7%. These findings indicate that no significant ion suppression or enhancement occurred under the applied experimental conditions. Detailed matrix effect data are presented in Table 4.

#### 3.3.5. Stability

The stability of exosome-containing plasma samples at low and high concentrations was assessed under various conditions, including storage in the autosampler for 24 h, exposure to room temperature for 4 h, storage at –40 °C for 7 days, and after three freeze–thaw cycles. Exosome concentrations were determined based on the peak areas of the three signature peptides (P1, P2, and P3). The results, summarized in Table 5, demonstrate that exosome plasma samples remained stable under all tested conditions, thereby confirming the robustness and reliability of the overall analytical method.

### 3.4. PK Studies of Exosomes in SD Rats

**Figure 3:**
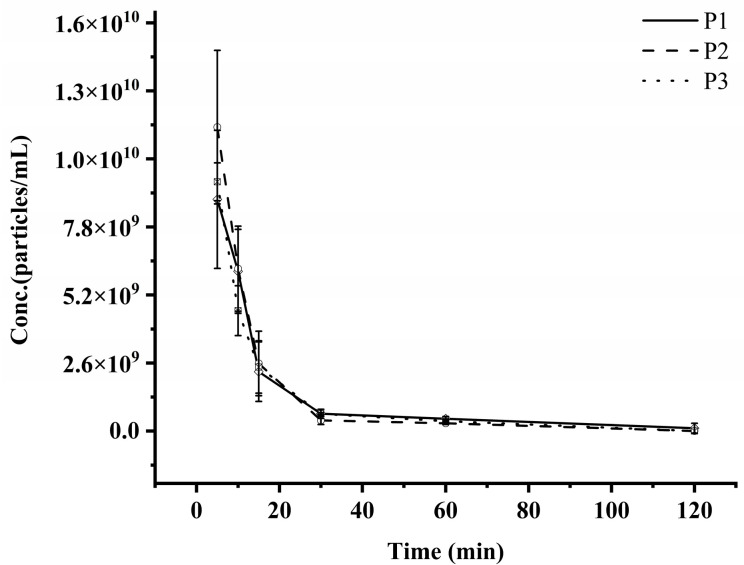
Concentration versus time profiles of exosomes in rat plasma, as determined by LC-MS/MS.

In this study, rat plasma samples collected following intravenous administration of exosomes were analyzed using LC-MS/MS. Exosome concentrations at each time point were calculated based on the peak areas of three signature peptides. The corresponding exosome concentration–time profile is illustrated in Figure 3, and the PK parameters are summarized in Table 6. Comparative analysis of the concentration–time curves and the PK parameters derived from the three signature peptides demonstrated consistent PK profiles for HEK 293F-derived exosomes in rats. The calculated half-life (T_1/2_) ranged from 10.67 to 13.64 min, maximum plasma concentration (C_max_) ranged from 8.85 × 10^9^ to 1.16 × 10^10^ particles/mL, and the area under the curve (AUC_0–t_) ranged from 1.52 × 10^10^ to 1.83 × 10^10^ min·particles/mL. The volume of distribution (Vd) was estimated at 25.78 to 39.08 mL, and the clearance (CL) ranged from 1.65 to 1.93 mL/min. No statistically significant differences were observed in the PK parameters obtained from the three peptides, collectively supporting the reliability and robustness of the established method for exosome PK studies in rats.

## 4. Discussion

Given that our study focused on plasma-derived exosomes, selecting an optimal isolation method was crucial. We compared three exosome extraction techniques from plasma: UC, SEC, and IMA. While UC is suitable for large-volume samples, it leads to significant exosome loss during repeated centrifugation steps [29], making it less optimal for our limited plasma volumes. IMA, which captures exosomes via antigen–antibody binding to specific surface markers, yields low quantities due to target-dependent capture and suffers from non-specific antibody binding [30]. In contrast, SEC separates exosomes based on particle size differences, resulting in minimal loss and superior purity compared to other methods [31]. Therefore, SEC was prioritized for its balance of recovery efficiency and compatibility with downstream LC-MS/MS analysis. Although SEC preferentially enriches the exosome drug candidate, the exosomes isolated from rat plasma may still be contaminated with endogenous exosomes. However, the accuracy of our PK method does not rely solely on the specificity of the extraction step. The subsequent MRM analysis provides high analytical specificity by detecting signature peptides uniquely derived from the administered exosomes.

The MRM-based LC-MS/MS method was comprehensively validated, and all results confirmed its reliability and reproducibility. When applied to post-dose rat plasma samples, the method revealed consistent mean exosome concentration–time profiles across the three signature peptides, with no significant discrepancies in PK parameters. Key findings showed that unmodified, label-free natural exosomes undergo rapid systemic clearance in rats, consistent with prior reports [21,32,33]. The rapid elimination following intravenous administration may be attributed to phagocytic uptake by erythrocytes and immune cells (e.g., macrophages, monocytes, granulocytes, and dendritic cells) [34]. The PK parameters indicated apparent Vds of 39.08, 25.78, and 33.90 mL, respectively, suggesting extensive tissue distribution, likely due to organ-specific sequestration or penetration into peripheral compartments [33].

MRM-based LC-MS/MS methods are widely employed in drug studies for accurately quantifying plasma drug concentrations, typically using internal standard calibration. However, the lack of validated internal standards for exosome quantification introduces slight variability in PK parameters (e.g., AUC, C_max_) derived from the three signature peptides in this study. Future research should focus on developing exosome-specific internal standards and optimizing pre-analytical workflows to enhance the robustness of this method for translational applications. It should also be noted that this method is based on the assumption that the expression levels of the target proteins are consistent across batches. If variations occur in the expression levels, the calibration curve would need to be re-established to ensure accurate quantification. Since our aim was to develop a PK method applicable to future exosome drug candidates, batch-to-batch consistency in protein content is critical. From chemical manufacturing and control (CMC) and quality control perspectives, such consistency is essential to ensure reproducible drug performance.

## 5. Conclusions

In conclusion, this study developed and validated an analytical method in compliance with European Medicines Agency (EMA) guidelines for evaluating exosome PK in rats. Our findings highlight the feasibility of LC-MS/MS as a reliable and robust platform for label-free quantification of exosomes in plasma, avoiding potential artifacts linked to exogenous labeling or genetic modifications. With further refinements aimed at enhancing sensitivity and standardization, this approach holds significant promise as a versatile tool for advancing PK research in exosome-based therapeutics.

## Figures and Tables

**Table 1:** Summary table comparing total protein concentration and exosome particle counts obtained by different extraction methods (mean ± SD, *n* = 3).

Method	Conc. (μg/μL)	Particles (Number/mL)
UC	0.14 ± 0.01	2.67 × 10^10^ ± 7.18 × 10^9^
IMA	0.27 ± 0.02	2.60 × 10^10^ ± 9.74 × 10^9^
SEC	0.79 ± 0.02	1.98 × 10^11^ ± 6.11 × 10^9^

**Table 2:** Linearity results for LC-MS/MS-based quantification of exosomes in rat plasma.

Peptide	Analyze and Criticize	Y = ax + b		
		a	B	r
P1	1	9.2182 × 10^−5^	27,020.2134	0.9982
	2	9.8481 × 10^−5^	15,870.1352	0.9982
	3	1.0040 × 10^−4^	14,832.9315	0.9985
P2	1	8.5952 × 10^−5^	5599.2859	0.9918
	2	8.1635 × 10^−5^	20,717.2711	0.9962
	3	8.6562 × 10^−5^	13,429.7681	0.9955
P3	1	3.5610 × 10^−5^	48,006.4672	0.9993
	2	4.0018 × 10^−5^	39,017.6424	0.9987
	3	3.9856 × 10^−5^	43,450.0808	0.9980

**Table 3:** Accuracy and precision results for LC-MS/MS-based quantification of exosomes in rat plasma.

Peptide	Sample Type	Intra-Day (*n* = 6)			Inter-Day (*n* = 18)		
		Mean Conc. (Particles/mL)	CV%	RE%	Mean Conc. (Particles/mL)	CV%	RE%
P1	LLOQ	2.94 × 10^8^	4.58	−2.05	2.76 × 10^8^	5.89	−8.05
	LQC	9.89 × 10^8^	5.44	9.83	9.76 × 10^8^	3.75	8.48
	MQC	5.80 × 10^9^	3.15	−3.34	5.56 × 10^9^	3.10	−7.37
	HQC	4.70 × 10^10^	6.98	−5.96	4.77 × 10^10^	5.08	−4.59
P2	LLOQ	3.35 × 10^8^	9.10	11.60	3.45 × 10^8^	2.82	15.15
	LQC	9.60 × 10^8^	3.24	6.70	8.78 × 10^8^	6.61	−2.43
	MQC	5.57 × 10^9^	5.12	−7.15	5.81 × 10^9^	9.32	−3.17
	HQC	4.59 × 10^10^	5.41	−8.29	4.78 × 10^10^	7.14	−4.36
P3	LLOQ	1.55 × 10^8^	6.73	3.66	1.60 × 10^8^	4.09	6.70
	LQC	2.73 × 10^8^	11.24	−8.94	3.17 × 10^8^	9.79	5.67
	MQC	6.32 × 10^9^	6.19	5.34	6.24 × 10^9^	5.76	3.98
	HQC	4.70 × 10^10^	3.50	−6.03	4.78 × 10^10^	9.75	−4.33

**Table 4:** Extraction recovery rates and matrix effects of exosome particle concentrations in rat plasma, calculated based on peak areas of three protein-specific signature peptides (mean ± SD, *n* = 3).

	Concentration	P1	P2	P3
Extraction Recovery (%)	Low	89.2 ± 3.9	90.7 ± 7.1	89.0 ± 6.6
	Mid	90.6 ± 9.9	98.9 ± 6.1	93.0 ± 3.1
	High	97.5 ± 7.9	86.6 ± 3.6	94.4 ± 7.9
Matrix Effect (%)	Low	89.1 ± 5.8	96.0 ± 8.0	94.1 ± 5.2
	Mid	98.6 ± 12.2	105.1 ± 12.7	97.1 ± 9.6
	High	96.5 ± 6.3	108.6 ± 1.4	102.7 ± 7.5

**Table 5:** Stability of exosome particle concentrations in rat plasma, calculated based on peak areas of three protein-specific signature peptides (mean ± SD, *n* = 3).

	Processing Conditions	Nominal Conc. (Particles/mL)	Mean Conc. (Particles/mL)	RSD%
P1	Autosampler for 24 h	5.00 × 10^10^	5.31 × 10^10^ ± 1.67 × 10^9^	3.15
		9.00 × 10^8^	8.74 × 10^8^ ± 5.48 × 10^7^	6.27
	Three freeze–thaw cycles	5.00 × 10^10^	4.65 × 10^10^ ± 4.62 × 10^9^	9.93
		9.00 × 10^8^	8.89 × 10^8^ ± 2.25 × 10^7^	2.53
	RT for 4 h	5.00 × 10^10^	4.48 × 10^10^ ± 6.16 × 10^9^	13.75
		9.00 × 10^8^	9.32 × 10^8^ ± 6.76 × 10^7^	7.25
	−40 °C for 7 d	5.00 × 10^10^	4.50 × 10^10^ ± 6.44 × 10^9^	14.33
		9.00 × 10^8^	8.73 × 10^8^ ± 6.70 × 10^7^	7.67
P2	Autosampler for 24 h	5.00 × 10^10^	4.76 × 10^10^ ± 1.76 × 10^9^	3.70
		9.00 × 10^8^	9.64 × 10^8^ ± 6.03 × 10^7^	6.26
	Three freeze–thaw cycles	5.00 × 10^10^	4.93 × 10^10^ ± 6.41 × 10^9^	12.99
		9.00 × 10^8^	9.49 × 10^8^ ± 1.11 × 10^8^	11.72
	RT for 4 h	5.00 × 10^10^	5.21 × 10^10^ ± 4.10 × 10^9^	7.88
		9.00 × 10^8^	8.61 × 10^8^ ± 3.65 × 10^7^	4.24
	−40 °C for 7 d	5.00 × 10^10^	5.33 × 10^10^ ± 3.96 × 10^9^	7.43
		9.00 × 10^8^	8.84 × 10^8^ ± 7.50 × 10^7^	8.49
P3	Autosampler for 24 h	5.00 × 10^10^	4.39 × 10^10^ ± 2.60 × 10^9^	5.91
		3.00 × 10^8^	3.23 × 10^8^ ± 2.22 × 10^7^	6.87
	Three freeze–thaw cycles	5.00 × 10^10^	5.29 × 10^10^ ± 4.77 × 10^9^	9.02
		3.00 × 10^8^	2.70 × 10^8^ ± 1.05 × 10^6^	0.39
	RT for 4 h	5.00 × 10^10^	5.06 × 10^10^ ± 5.67 × 10^9^	11.20
		3.00 × 10^8^	2.72 × 10^8^ ± 3.33 × 10^7^	12.26
	−40 °C for 7 d	5.00 × 10^10^	4.44 × 10^10^ ± 2.77 × 10^9^	6.24
		3.00 × 10^8^	3.22 × 10^8^ ± 1.79 × 10^7^	5.56

**Table 6:** Pharmacokinetic parameters of plasma from three rats based on drug concentration data of exosomes, as determined by LC-MS/MS (mean ± SD, *n* = 3).

Parameters	Units	P1	P2	P3
T_1/2_	min	13.64 ± 2.10	10.67 ± 0.90	13.52 ± 2.11
T_max_	min	5 ± 0	5 ± 0	5 ± 0
C_max_	particles/mL	8.85 ± 2.15 × 10^9^	1.16 ± 0.24 × 10^10^	9.53 ± 0.59 × 10^9^
AUC_0–t_	(min × particles/mL)	1.52 ± 0.33 × 10^11^	1.83 ± 0.37 × 10^11^	1.66 ± 0.18 × 10^11^
MRT_0–t_	min	16.16 ± 3.76	10.11 ± 1.11	12.70 ± 1.09
Vd	mL	39.08 ± 13.50	25.78 ± 6.14	33.90 ± 4.44
CL	mL/min	1.93 ± 0.38	1.65 ± 0.31	1.75 ± 0.21

## Data Availability

The data presented in this study are available within the article and Supplementary Materials.

## Supplementary Materials

The following supporting information can be downloaded at: https://www.mdpi.com/article/10.3390/pharmaceutics17060699/s1, Figure S1: Characterization of HEK 293F-derived exosomes; Table S1: The peptide sequences confirmed as unique by BLAST analysis; Table S2: LC-MS/MS detection information and results of prototypic peptides.
