# Supplementary material for: A Label-Free Liquid Chromatography–Tandem Mass Spectrometry Method for the Quantitative Analysis of Exosome Pharmacokinetics In Vivo
Source: Pharmaceutics. 2025 May 27;17(6):699. doi: 10.3390/pharmaceutics17060699 (PMC12195665; doi:10.3390/pharmaceutics17060699)
Supplement: Supplementary file 1 [file pharmaceutics-17-00699-s001.zip › pharmaceutics-3629959-supplementary.pdf]

Supplementary Materials

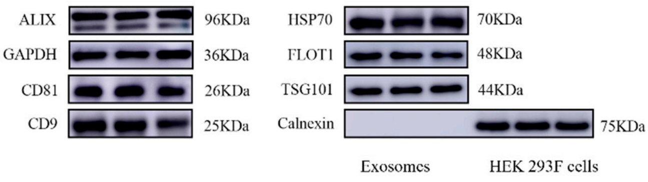

A

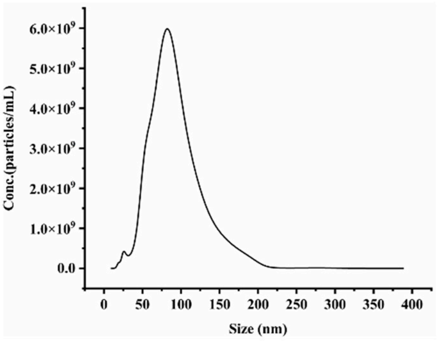

B

Supplementary Figure S1. Characterization of HEK 293F-derived exosomes. (A) Western blot analysis of exosomal marker proteins, including CD9, CD81, TSG101, FLOT1, HSP70, and ALIX, while Calnexin were absent (B) Particle concentration profile determined by NTA

Supplementary Table S1. The peptide sequences confirmed as unique by BLAST analysis.

Protein	Peptide
ALIX	RAAVGRPLDKHEGALETLLR
	LANQAADYFGDAFKQCQYKDTLPK
	QCQYKDTLPK
	QCQYKDTLPKEVFPVLAAK
	DTLPKEVFPVLAAK
	HCIMQANAEYHQSILAKQQK
	LQHAAELIKTVASR
	TVASRYDEYVNVK
	VPDLKDLDPIGKATLVK
	DLDPIGKATLVK
	DLDPIGKATLVKSTPVNVPISQK
	STPVNVPISQKFTDLFEK
	MVPVSVQQSLAAYNQR
	SRSVIEQGGIQTVDQLIK
	VYGGLTTKVQESLK
	MKQSNNEANLR
	TMPPTKPQPPARPPPPVLPANR
	TMQGSEVVNVLKSLLSNLDEVKK
CD9	MPVKGGTKCIK
	DEVIKEVQEFYKDTYNK
	EVQEFYKDTYNK
	TKDEPQRETLK
	DVLETFTVKSCPDAIK
	SCPDAIKEVFDNK

Continued Supplementary Table S1. The peptide sequences confirmed as unique by BLAST analysis.

Protein	Peptide
CD63	QQMENYPKNNHTASILDRMQADFK
	NNHTASILDR
	NRVPDSCCINVTVGCGINFNEK
	VPDSCCINVTVGCGINFNEK
	EGCVEKIGGWLR
	EGCVEKIGGWLRK
Cofilin-1	MASGVAVSDGVIKVFNDMK
	VRKSSTPEEVK
	SSTPEEVKK
	EILVGDVGQTVDDPYATFVK
	YALYDATYETK
	NIILEEGK
	KEDLVFIFWAPESAPLK
	YALYDATYETKESK
CHMP6	QSRVTEQDKAILQLK
	MHQVMSIEEVER
	KRYQEQLLDR
	ARPRQAELVAAS
FLOT1	QEKVSAQYLSEIEMAK
	VSAQYLSEIEMAK
	VSAQYLSEIEMAKAQR
	DYELKKAAYDIEVNTR
	AAYDIEVNTR
	LAEAEKSQLIMQAEAEAASVRMR
	SQLIMQAEAEAASVR
	MRGEAEAFAIGAR
	LTGVSISQVNHKPLRTA
GAPDH	FHGTVKAENGK
	LVINGNPITIFQER
	WGDAGAEYVVESTGVFTTMEK
	AGAHLQGGAK
	GALQNIIPASTGAAK
	VIPELNGKLTGMAFR
	LISWYDNEFGYSNR
FLOT2	DQFAKLVR
	DAGIREAECKK
CD81	DVKQFYDQALQQAVVDDDANNAK
	TFHETLDCCGSSTLTALTTSVLK
	QFYDQALQQAVVDDDANNAK
	EDCHQKIDDLFSGK
	NNLCPSGSNIISNLFK

Supplementary Table S2 LC-MS/MS detection information and results of prototypic peptide

Protein	Peptide	Retention Time	Precursor ion	Product ion	Area
ALIX	DTLPKEVFPVLAAK	45.01	764.44	1126.61	1.76×10^5
				563.81	6.76×10^5
				598.39	1.57×10^5
	HCIMQANAEYHQSILAKQQK	34.85	800.06	1218.50	3.38×10^5
				609.76	1.46×10^5
				301.68	2.02×10^5
	LQHAAELIKTVASR	50.91	768.95	661.40	4.33×10^5
				533.30	6.57×10^5
				432.26	1.17×10^5
	DLDPIGKATLVK	29.87	635.37	460.31	1.05×10^5
				330.23	3.98×10^5
				230.66	8.17×10^5
	STPVNVPISQKFTDLFEK	32.82	684.03	757.90	2.00×10^5
				270.16	1.39×10^5
				384.55	1.16×10^5
	TMQGSEVVNVLKSLLSNLDEVKK	36.38	844.46	949.57	5.33×10^6
				623.36	3.60×10^6
				244.48	2.96×10^6
CD9	MPVKGGTKCIK	15.34	406.89	456.26	6.68×10^4
				570.31	9.97×10^6
				285.66	7.14×10^5
	EVQEFYKDTYNK	38.51	782.37	357.18	4.00×10^6
				570.76	3.04×10^5
				147.11	2.40×10^5
	DVLETFTVKSCPDAIK	40.44	911.97	215.10	2.80×10^4
				328.19	5.00×10^4
				260.20	3.91×10^5
FLOT1	VSAQYLSEIEMAK	24.90	490.25	651.31	9.36×10^5
				496.26	4.46×10^5
				478.23	9.16×10^6
	AAYDIEVNTR	30.79	384.53	663.30	5.95×10^5
				332.15	2.85×10^5
				245.14	7.24×10^5
	LAEAEKSQLIMQAEAEAASVRMR	36.38	844.77	385.21	2.78×10^6
				807.41	4.51×10^6
				460.24	5.12×10^6
	LTGVSISQVNHKPLRTA	29.06	607.68	458.26	2.66×10^6
				681.38	1.88×10^6
				682.39	4.43×10^6
	QEKVSAQYLSEIEMAK	53.20	927.47	1047.55	2.52×10^6
				243.14	4.28×10^6
				591.32	3.01×10^6

Continued Supplementary Table S2 LC-MS/MS detection information and results of prototypic peptide

Protein	Peptide	Retention Time	Precursor ion	Product ion	Area
CD63	NRVPDSCCINVTVGCGINFNEK	47.48	851.73	651.31	9.36×10^5
				537.27	4.85×10^5
				276.16	3.77×10^5
	VPDSCCINVTVGCGINFNEK	38.43	761.68	312.16	8.21×10^5
				200.10	9.28×10^5
				816.86	1.99×10^6
	EGCVEKIGGWLR	24.99	468.57	347.10	2.22×10^6
				288.20	1.51×10^4
				234.47	1.18×10^6
	EGCVEKIGGWLRK	31.24	766.41	446.17	2.26×10^4
				408.70	3.39×10^4
				330.20	2.44×10^4
TSG101	YRDLTVRETVNVITLYK	38.86	695.06	949.57	1.86×10^5
				524.31	2.73×10^5
				310.18	5.43×10^5
	DLTVRETVNVITLYK	48.61	882.50	229.12	8.92×10^5
				585.34	4.93×10^5
				368.73	2.86×10^5
	ETVNVITLYK	40.51	590.33	757.41	4.67×10^6
				870.49	3.58×10^6
				328.68	8.27×10^5
	IYLPYLHEWK	33.89	454.58	277.15	1.74×10^5
				390.24	2.65×10^5
				147.11	8.70×10^4
	MKEEMDRAQAELNALK	33.27	938.97	260.14	9.80×10^4
				764.30	5.36×10^5
				260.20	1.27×10^5
	AQAELNALK	37.00	479.27	698.35	7.13×10^5
				349.68	1.12×10^6
				147.11	9.34×10^5
Cofilin-1	YALYDATYETK	43.03	669.32	235.11	6.95×10^5
				348.19	1.51×10^5
				827.38	2.13×10^5
	YALYDATYETKESK	25.54	561.27	697.32	2.72×10^5
				348.19	2.03×10^5
				985.48	1.09×10^5
	KEDLVFIFWAPESAPLK	48.98	664.03	845.48	2.53×10^5
				741.41	5.56×10^5
				371.21	2.43×10^5

Continued Supplementary Table S2 LC-MS/MS detection information and results of prototypic peptide

Protein	Peptide	Retention Time	Precursor ion	Product ion	Area
CD81	DVKQFYDQALQQAVVDDDANNAK	50.17	865.75	896.41	3.42×10^5
				548.26	3.38×10^5
				850.42	1.78×10^5
	TFHETLDCCGSSTLTALTTSVLK	31.78	848.08	537.23	8.44×10^5
				441.26	3.35×10^5
				311.86	1.83×10^5
HSP70	AAAIGIDLGTTYSCVGVFQHGK	38.51	755.72	143.08	4.04×10^6
				214.12	1.05×10^7
				884.92	4.32×10^6
	HWPFQVINDGDKPK	25.51	560.95	324.15	2.64×10^6
				795.39	5.37×10^6
				348.67	3.42×10^6
	AFYPEEISSMVLTK	42.07	807.91	219.11	1.18×10^7
				1007.54	3.93×10^6
				698.86	1.71×10^7
	QTQIFTTYSDNQPGVLIQVYEGER	43.59	929.46	1106.58	2.61×10^6
				880.42	4.00×10^6
				752.36	2.32×10^6
